# *BRAF* ^*V600E*^ expression in histiocytic sarcoma associated with splenic marginal zone lymphoma: a case report

**DOI:** 10.1186/s13256-017-1253-z

**Published:** 2017-04-05

**Authors:** John L. Vaughn, C. Eric Freitag, Jessica A. Hemminger, Jeffrey A. Jones

**Affiliations:** 1 Department of Internal Medicine, The Ohio State University, Columbus, OH USA; 2 Department of Pathology, The Ohio State University, Columbus, OH USA; 3 Division of Hematology, Department of Internal Medicine, The Ohio State University, Columbus, OH USA

**Keywords:** Histiocytic sarcoma, Marginal zone B-cell lymphoma, Proto-oncogene proteins B-raf, Immunohistochemistry

## Abstract

**Background:**

Histiocytic sarcoma is a rare histiocytic neoplasm of unknown etiology that constitutes less than 1% of hematologic malignancies. A few cases of histiocytic sarcoma harboring the *BRAF* ^*V600E*^ mutation have been reported, but this finding has not been confirmed in all studies.

**Case presentation:**

We report the case of a 63-year-old white woman with a history of splenic marginal zone lymphoma who presented with 2 weeks of right-sided neck swelling. Positron emission tomography revealed an intensely hypermetabolic and destructive soft tissue mass in her right skull base. A bone marrow biopsy was performed, which revealed an infiltrate of malignant cells characterized as large pleomorphic cells with frequent folded/irregular nuclei, variably prominent nucleoli, fine chromatin, and abundant amounts of eosinophilic cytoplasm. The malignant cells were positive for CD163, CD68 (granular), lysozyme (granular), CD4, and CD45 (partial). Based on the biopsy findings, she was diagnosed as having histiocytic sarcoma. The malignant cells tested positive for the BRAF^V600E^ protein using immunohistochemistry. Before treatment of her histiocytic sarcoma could be initiated, she developed disseminated intravascular coagulation and acute hypoxemic respiratory failure secondary to non-cardiogenic pulmonary edema. She decided to pursue comfort care and died in our hospital 2 weeks following admission.

**Conclusions:**

Our case illustrates the aggressive nature of histiocytic sarcoma, and provides rare evidence that histiocytic sarcoma associated with indolent lymphomas may harbor the *BRAF* ^*V600E*^ mutation. Further research is needed to clarify the role of targeted therapies such as vemurafenib in the treatment of patients with this disorder.

## Background

Histiocytic sarcoma (HS) is a rare histiocytic neoplasm of unknown etiology that constitutes less than 1% of hematologic malignancies. HS can occur as a primary disorder or as a secondary disorder arising from other hematologic malignancies [1]. HS can affect any organ including skin, bone, soft tissues, gastrointestinal tract, and central nervous system. The histology of HS is characterized by large pleomorphic cells with eosinophilic cytoplasm. The cells typically express the histiocytic markers CD68, CD163, and lysozyme and lack lymphoid, myeloid, Langerhans, and follicular dendritic cell markers [2]. HS follows an aggressive clinical course and responds poorly to treatment, which makes managing patients with this disorder challenging [3]. However, a small number of recent case reports have demonstrated the presence of the *BRAF* ^*V600E*^ mutation in HS, and the presence of this mutation raises the possibility of using targeted therapies such as vemurafenib [4–6]. We report the case of a patient with splenic marginal zone lymphoma (SMZL) who developed HS expressing the BRAF^V600E^ protein.

## Case presentation

**Fig. 1:**
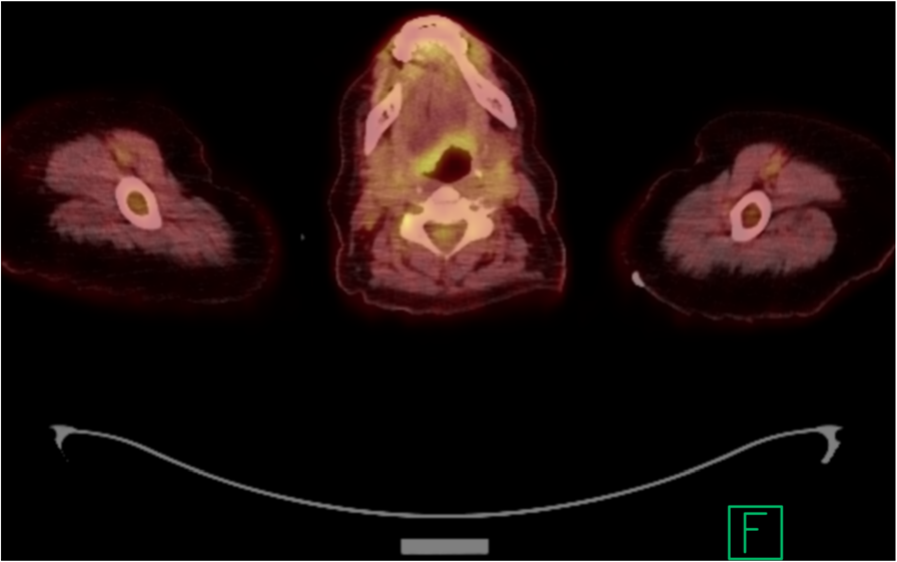
Positron emission tomography scan demonstrating hypermetabolic soft tissue fullness at the right tongue base with a maximum standardized uptake value of 4.5

**Fig. 2:**
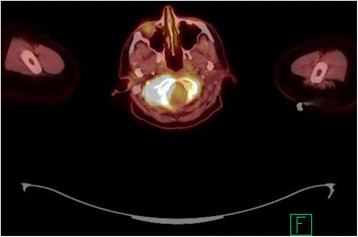
Positron emission tomography scan demonstrating an intensely hypermetabolic and destructive soft tissue mass in the right skull base with a maximum standardized uptake value of 31.6

A 63-year-old white woman with a history of SMZL diagnosed in 2014 and treated with splenectomy presented to an outside hospital with 2 weeks of right-sided neck swelling. A computed tomography scan of her neck revealed asymmetric soft tissue fullness posteriorly in her tongue on the right side, which suggested a possible infectious or inflammatory process involving her tongue. She was given a single dose of ampicillin-sulbactam intravenously and transferred to our emergency department for further evaluation. After evaluation by an otolaryngologist, she was prescribed a 10-day course of amoxicillin-clavulanate for lingual tonsillitis and discharged. She returned 1 week later with fevers, dysphagia, and worsening pain in her neck. She was admitted to our hospital and underwent a positron emission tomography (PET) scan, which demonstrated hypermetabolic soft tissue fullness at her right tongue base with extension into the vallecula and a maximum standardized uptake value (SUV) of 4.5 (Fig. 1). The PET scan also showed an intensely hypermetabolic and destructive soft tissue mass in her right skull base with a maximum SUV of 31.6 (Fig. 2).

**Fig. 3:**
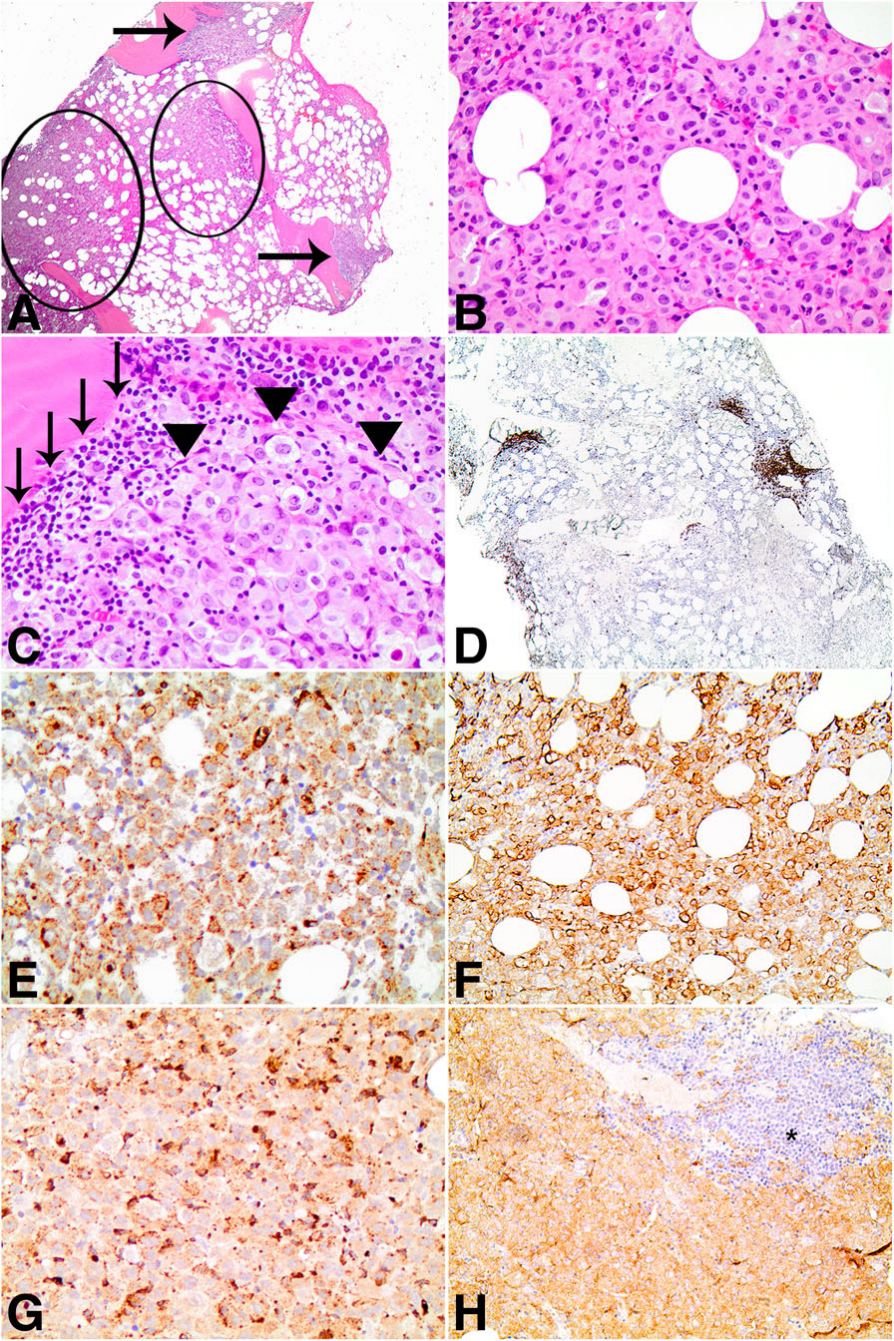
**a** Bone marrow biopsy with patchy involvement by histiocytic sarcoma (*circles*) as well as small paratrabecular lymphoid aggregates (*arrows*). Hematoxylin and eosin, ×40. **b** Histiocytic sarcoma comprised large pleomorphic cells with frequent folded/irregular nuclei and abundant amounts of eosinophilic cytoplasm. Hematoxylin and eosin, ×400. **c** Histiocytic sarcoma (*arrowheads*) and adjacent small paratrabecular lymphoid aggregate (*arrows*) composed of small lymphocytes. Hematoxylin and eosin, ×400. **d** The atypical paratrabecular B cell-rich lymphoid aggregates are highlighted by CD20 immunohistochemistry (×40). The histiocytic sarcoma is positive for **e** CD68 (×400), **f** CD163 (×200), **g** lysozyme (×400), and **h** BRAF^V600E^ (×200), as determined by immunohistochemistry. **h** An atypical paratrabecular lymphoid aggregate (*asterisk*) is negative for BRAF^V600E^ immunohistochemistry

Given the presence of a mass in her right skull base, a lumbar puncture was performed, which revealed rare, large mononuclear cells suggestive of atypical monocytes/macrophages. A subsequent bone marrow biopsy contained a neoplastic infiltrate of large pleomorphic cells with frequent folded/irregular nuclei, variably prominent nucleoli, fine chromatin, and abundant amounts of eosinophilic cytoplasm (Fig. 3a, b). A few multinucleated neoplastic cells were noted. The malignant cells were positive for CD68 (granular; Fig. 3e), CD163 (Fig. 3f), lysozyme (granular; Fig. 3g), CD4, CD45 (partial, weak), and S-100 (variable) and negative for CD20, PAX5, CD3, AE1/AE3, SOX10, CD43, ALK, CD30, granzyme, CD2, CD56, MPO, CD1a, CD21, CD35, HMB-45, MELAN-A, EBER, and CD138. The Ki67 proliferation index in the neoplastic infiltrate was at least 90%. Based on the biopsy findings, our patient was diagnosed as having HS. After the diagnosis of HS was made, the cells were tested for the BRAF^V600E^ protein using immunohistochemistry (clone VE1; Spring Bioscience, Pleasanton, CA, USA). The HS cells stained positive (Fig. 3h), which indicated expression of the BRAF^V600E^ protein. The presence of an underlying *BRAF* ^*V600E*^ mutation was not confirmed with deoxyribonucleic acid (DNA) sequencing.

In addition to HS, her bone marrow biopsy contained multiple atypical paratrabecular and nonparatrabecular B cell-rich lymphoid aggregates composed of small, mature lymphocytes as highlighted by CD20, PAX5, and CD3 immunohistochemistry (Fig. 3c, d). Although flow cytometry was negative for a clonal B cell population, the atypical lymphoid aggregates seemed to indicate low level bone marrow involvement by her known SMZL. The atypical lymphoid aggregates were negative for BRAF^V600E^ immunohistochemistry (Fig. 3h). Following her bone marrow biopsy, a needle biopsy of the soft tissue mass in her right skull base was planned. However, she was diagnosed as having disseminated intravascular coagulation and the biopsy was postponed indefinitely. Her laboratory studies at that time were significant for a prothrombin time (PT) of 48.3 seconds, partial thromboplastin time (PTT) of 93 seconds, international normalized ratio (INR) of 5.3, fibrinogen of 167 mg/dL, platelet count of 35 K/μL, and schistocytes on her peripheral blood smear. Before treatment of HS could be initiated, she developed acute hypoxemic respiratory failure secondary to non-cardiogenic pulmonary edema. She was given high-dose steroids with no improvement in her status. Over the next several days, her respiratory status continued to worsen, and she decided to pursue comfort care. She died in our hospital 2 weeks following admission. Table 1 illustrates the timeline of her illness.

**Table 1:** Timeline of histiocytic sarcoma

Event	Time point
First onset of symptoms	Day 0
Initial presentation to emergency department	Day 14
Admission to hospital	Day 21
Bone marrow biopsy	Day 26
Death	Day 36

## Discussion

The finding of BRAF^V600E^ expression in our patient is significant. To date, few studies have investigated the presence of the *BRAF* ^*V600E*^ mutation in HS, and the results have been mixed. A study by Haroche and colleagues in 2012 found a high prevalence of the mutation in Erdheim–Chester disease but not in other non-Langerhans cell histiocytoses (0 out of three cases of HS possessed the mutation) [7]. Another study by Bubolz and colleagues concluded that *BRAF* mutations in histiocytic neoplasms are restricted to the Langerhans-cell type, although HS was not included in the study [8]. In contrast, a study by Go and colleagues in 2014 detected the mutation in five out of eight (62.5%) cases of HS studied [4]. The presence of the mutation has also been documented in two other cases: one case of primary HS involving the central nervous system, and one case of HS arising from hairy cell leukemia [5, 6]. Our case provides rare evidence that HS associated with indolent lymphomas may harbor the *BRAF* ^*V600E*^ mutation.

Our case also illustrates the aggressive nature of HS. Despite obtaining a diagnosis within 2 weeks of admission, our patient declined rapidly and was unable to receive chemotherapy. It is unclear whether obtaining a diagnosis sooner would have improved her outcome since HS is known for being resistant to chemotherapy, and the best chemotherapy regimen is unknown [3]. Furthermore, our patient had evidence of bone marrow and central nervous system involvement, and multifocal HS carries a worse prognosis than unifocal disease [9]. Given the presence of BRAF^V600E^ expression, we may have utilized vemurafenib in addition to chemotherapy. However, further research is needed to clarify the role of targeted therapies such as vemurafenib in the treatment of patients with this disorder.

## Conclusions

Our case illustrates the aggressive nature of HS, and provides rare evidence that HS associated with indolent lymphomas may harbor the *BRAF* ^*V600E*^ mutation. Further research is needed to clarify the role of targeted therapies such as vemurafenib in the treatment of patients with this disorder.

## Abbreviations

HS: Histiocytic sarcoma
PET: Positron emission tomography
SMZL: Splenic marginal zone lymphoma
SUV: Standardized uptake value
